# A population-based comparison of second primary cancers in Germany and Sweden between 1997 and 2006: clinical implications and etiologic aspects

**DOI:** 10.1002/cam4.116

**Published:** 2013-08-27

**Authors:** Hao Liu, Kari Hemminki, Jan Sundquist, Bernd Holleczek, Alexander Katalinic, Katharina Emrich, Lina Jansen, Hermann Brenner

**Affiliations:** 1 Division of Molecular Genetic Epidemiology, German Cancer Research Center (DKFZ)Heidelberg, Germany; 2 Center for Primary Health Care Research, Lund UniversityMalmö, Sweden; 3 Stanford Prevention Research Center, Stanford University School of MedicinePalo Alto, California; 4 Saarland Cancer RegistrySaarbrücken, Germany; 5 Cancer Registry of Schleswig-HolsteinInstitute of Cancer Epidemiology, University of LübeckLübeck, Germany; 6 Cancer Registry of Rhineland-PalatinateInstitute of Medical Biostatistics, Epidemiology and InformaticsUniversity Medical Center, Johannes Gutenberg University MainzMainz, Germany; 7 Division of Clinical Epidemiology and Aging Research, German Cancer Research Center (DKFZ)Heidelberg, Germany

**Keywords:** Clinical implications, etiologic aspects, population-based comparison, second primary cancer

## Abstract

Second primary cancer (SPC) has become an increasing concern in cancer survivorship. Patterns of SPCs in different populations may offer clinical implications and research priorities into SPCs. This study is devoted to compare the occurrences and rank correlations of SPCs between Germany and Sweden. Patients diagnosed with 10 common first primaries between 1997 and 2006 from the Swedish Family-Cancer Database and 10 German cancer registries were included in this population-based study. Spearman's rank correlation coefficients were used to evaluate the strength of the relationship of SPCs between the German and Swedish datasets. Spearman's rank correlation coefficients suggested a strong positive correlation between the German and Swedish datasets based on the ranks of thirty possible SPCs after all selected first cancers. This was also true when we compared the rankings and proportions of the five most common SPCs after site-specific first primaries between the two populations. For kidney cancer, non-Hodgkin's lymphoma, and leukemia the components of the five most common SPCs was exactly the same. Also, the ranking and the proportions for the three most common SPCs (i.e., colorectal, bladder, and lung cancers) after prostate cancer were identical in the two populations, as were those after most other primary cancers. The strikingly consistent patterns of SPCs in the two populations provide excellent opportunities for joint studies and they also suggest that many underlying reasons for SPC may have universal and tangible causes that await mechanistic dissection.

The strikingly consistent patterns of second primary cancers (SPCs) in German and Swedish populations provide excellent opportunities for joint studies and they also suggest that many underlying reasons for SPC may have universal and tangible causes that await mechanistic dissection.

## Introduction

Second primary cancer (SPC) is one of the major prognostic factors among cancer survivors and it is estimated to be the sixth most common form of malignancy in the world 1. Besides this general sense of priority, there should be a special emphasis on SPC research for various reasons, including the possibility of reducing risk of SPC through control of the relevant environmental, behavioral, and genetic factors, once these have been identified 2,3. Study of SPC will be rewarding if the scope and the strategies are properly set 4. SPCs can be grouped according to major identified etiologies, such as syndromic, treatment related, and shared exposure related 3. Carcinogenesis of SPC is a complex process 2,5, which may be caused by interactions between treatment for the first cancer, the genetic predisposition, as well as environmental and behavioral factors. Nielsen and colleagues found that heterogeneity in the risk of SPC was substantial across cancer types, pointing out the need to consider the pairs of first and second cancers rather than the overall risk 6. Monitoring of the associations of particular first and SPCs in different populations may reveal novel carcinogenic pathways relevant to SPC, which may result in clinical practice improvement.

Many multicenter studies on SPCs have been conducted but the concordance in the occurrence of SPCs between various centers has rarely been reported. Obviously, the incidence of any primary cancers is related to the incidence of SPCs 7. The present study is devoted to the comparison of the occurrence and ranking of specific SPC after 10 common first primaries between Germany and Sweden by using population-based cancer registries. Even though the overall cancer incidence is not very different between Sweden and the German cancer registries, rates of individual cancers differ, most notably for high rates in lung- and smoking-related cancers in German men, high prostate cancer rates in Swedish men, and somewhat higher colorectal cancer (CRC) rates among German men and women (Cancer Incidence in Five Continents IX, http://www.iarc.fr).

## Patients and Methods

The present study used the data from two sources. One is the Swedish Family-Cancer Database which includes data on 12.1 million individuals in the latest update of the year 2008, and over 1.1 million cancer patients, retrieved from the Swedish Cancer Registry (1958–2008) 8. The other source is the pooled dataset from 11 German cancer registries including data on 1.1 million cancer patients diagnosed between 1997 and 2006, covering a population of 33 million people 9. The present study included 10 of these Germany cancer registries. For comparisons, the present study thus included only the cancer patients diagnosed during 1997–2006 from the two datasets. An ad hoc study of the diagnostic accuracy of second cancers in the Swedish Cancer Registry indicated that 98% were correctly classified 10, and the German cancer registries strictly followed the multiple primary coding rules of the International Agency for Research on Cancer (IARC). In the Swedish Cancer Registry, the basic coding of tumor site has been performed according to ICD-O/2 (1993–2004) and ICD-O/3 (2005-w) and the different classifications are translated to ICD-7 to enable longer trends (codes: 140-197, 200-209). In the German dataset, tumors were identified based on ICD-O/3 and later converted into ICD-10 (codes: C00-C75, C81-C96). The case numbers of the 10 common first primary cancers for the German and Swedish datasets are shown in Table 1. The SPCs were grouped into thirty major types in present study.

**Table:** Case numbers of selected 10 first cancers in Germany and Sweden (1997–2006)

First cancer sites	Germany	Sweden
Colorectum	149,038	38,789
Lung	98,856	24,953
Breast	149,471	51,691
Endometrium	28,215	10,373
Prostate	126,928	66,057
Kidney	35,900	7545
Urinary bladder	41,274	15,963
Melanoma	33,194	14,703
NHL	29,034	11,592
Leukemia	20,058	10,504

NHL, non-Hodgkin's lymphoma.

Spearman's rank correlation coefficients were used to test the strength of the relationship of the ranks of thirty possible SPCs after site-specific first primaries between the German and Swedish datasets. All the analyses were carried out by using SAS software version 9.2. The study was approved by the local ethics committees and the recommendations of Declaration of Helsinki had been followed.

## Results

Table 2 provides a summary of the five most common SPCs following 10 common first cancers in Germany and Sweden. The five leading SPCs after kidney cancer were identical between the two datasets, including prostate, colorectal, urinary bladder, lung, and breast cancers. For non-Hodgkin's lymphoma (NHL) and leukemia, the same order of the five SPCs was also observed in the two datasets. In addition, the three most common SPCs after prostate cancer, ranked in descending prevalence, were CRC (20.9% in Germany vs. 23.7% in Sweden), bladder cancer (20.0% vs. 17.8%), and lung cancer (14.3% vs. 12.3%) (Table 2). For the remaining first cancers, four of the five leading SPCs were identical in the two populations (Table 2). For instance, the four SPCs after CRC were prostate, lung, breast, and kidney cancers. For lung cancer, the SPCs were the tumors of prostate, colorectum, urinary bladder, and upper aerodigestive tract. For breast cancer, the SPCs were colorectal, endometrial, lung, and ovary cancers. Breast cancer, ovary cancer, CRC, and lung cancers were the four SPCs after endometrial cancer. Bladder cancer was followed by the four SPCs including prostate cancer, lung cancer, CRC, and kidney cancer. Prostate cancer (18.6% vs. 29.3%), breast cancer (14.4% vs. 15.6%), CRC (13.8% vs. 12.3%), and lung cancer (9.1% vs. 6.3%) following melanoma were found for the two datasets.

**Table:** Occurrence of the five most common SPCs and rank correlations of SPCs after 10 common first cancers between Germany and Sweden (1997–2006)

First cancers	Germany				Sweden							Germany vs. Sweden								
	Second cancers	*N*	%	Second cancers	*N*	%	*r*_s_^1^	*P*-value
Colorectum	Prostate	1050	20.1	Prostate	584	27.2		
	Lung	596	11.4	Breast	247	11.5		
	Breast	524	10.0	Lung	187	8.7		
	Kidney	444	8.5	Bladder	182	8.5		
	Stomach	335	6.4	Kidney	98	4.6	0.90	<0.001
Lung	Prostate	310	15.9	Prostate	116	21.0		
	Colorectum	256	13.1	Colorectum	75	13.6		
	UADT	249	12.8	Breast	61	11.1		
	Bladder	163	8.4	Bladder	53	9.6		
	Kidney	147	7.6	UADT	28	5.1	0.86	<0.001
Breast	Colorectum	674	19.8	Colorectum	392	19.0		
	Endometrium	490	14.4	Endometrium	269	13.0		
	Lung	296	8.7	Lung	241	11.7		
	Ovary	243	7.1	Ovary	130	6.3		
	Kidney	235	6.9	Melanoma	129	6.2	0.85	<0.001
Endometrium	Breast	391	27.7	Breast	215	28.2		
	Ovary	288	20.4	Colorectum	137	18.0		
	Colorectum	186	13.2	Ovary	106	13.9		
	Lung	74	5.2	Lung	41	5.4		
	Kidney	62	4.4	Leukemia	28	3.7	0.83	<0.001
Prostate	Colorectum	1188	20.9	Colorectum	963	23.7		
	Bladder	1139	20.0	Bladder	724	17.8		
	Lung	811	14.3	Lung	500	12.3		
	Kidney	570	10.0	Melanoma	240	5.9		
	Stomach	337	5.9	NHL	235	5.8	0.96	<0.001
Kidney	Prostate	598	28.4	Bladder	143	23.9		
	Colorectum	285	13.5	Prostate	143	23.9		
	Bladder	278	13.2	Colorectum	56	9.3		
	Lung	193	9.2	Lung	52	8.7		
	Breast	138	6.6	Breast	46	7.7	0.81	<0.001
Bladder	Prostate	2037	55.1	Prostate	844	45.4		
	Lung	446	12.1	Lung	212	11.4		
	Colorectum	318	8.6	Colorectum	174	9.4		
	Kidney	258	7.0	Kidney	153	8.2		
	Stomach	84	2.3	Breast	72	3.9	0.72	<0.001
Melanoma	Prostate	224	18.6	Prostate	272	29.3		
	Breast	174	14.4	Breast	145	15.6		
	Colorectum	167	13.8	Colorectum	114	12.3		
	Lung	110	9.1	Lung	58	6.3		
	Kidney	85	7.0	Bladder	45	4.8	0.85	<0.001
NHL	Lung	166	14.8	Prostate	138	20.3		
	Colorectum	152	13.6	Colorectum	90	13.3		
	Prostate	148	13.2	Lung	58	8.5		
	Breast	96	8.6	Leukemia	55	8.1		
	Leukemia	81	7.2	Breast	45	6.6	0.91	<0.001
Leukemia	Prostate	112	16.1	Prostate	111	19.5		
	Colorectum	99	14.2	Lung	74	13.0		
	Lung	98	14.1	Colorectum	72	12.7		
	NHL	76	10.9	NHL	42	7.4		
	Breast	50	7.2	Breast	40	7.0	0.90	<0.001

SPCs, second primary cancers; UADT, upper aerodigestive tract; NHL, non-Hodgkin's lymphoma.

Spearman's correlation coefficient (*r*_s_) was calculated based on the ranks of 30 possible SPCs after first cancers.

Spearman's rank correlation coefficients showed that there was a strong positive correlation based on the ranks of thirty possible SPCs after all selected first cancers between the two datasets. Spearman's correlation coefficient (*r*_s_) was 0.90 for CRC, 0.86 for lung cancer, 0.85 for breast cancer, 0.83 for endometrial cancer, 0.96 for prostate cancer, 0.81 for kidney cancer, 0.72 for bladder cancer, 0.85 for melanoma, 0.91 for NHL, and 0.90 for leukemia. *P* values for all these *r*_s_ were less than 0.001 (Table 2).

**Figure 1:**
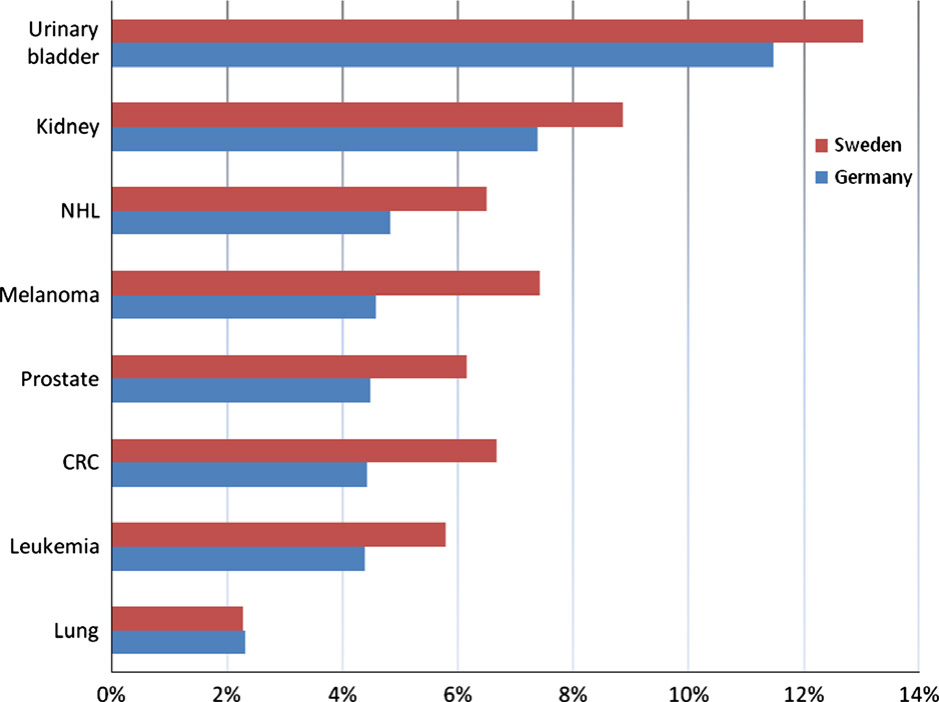
Percentage of all second primary cancers (SPCs) combined following 10 common first cancers in Germany and Sweden, male, 1997–2006.

**Figure 2:**
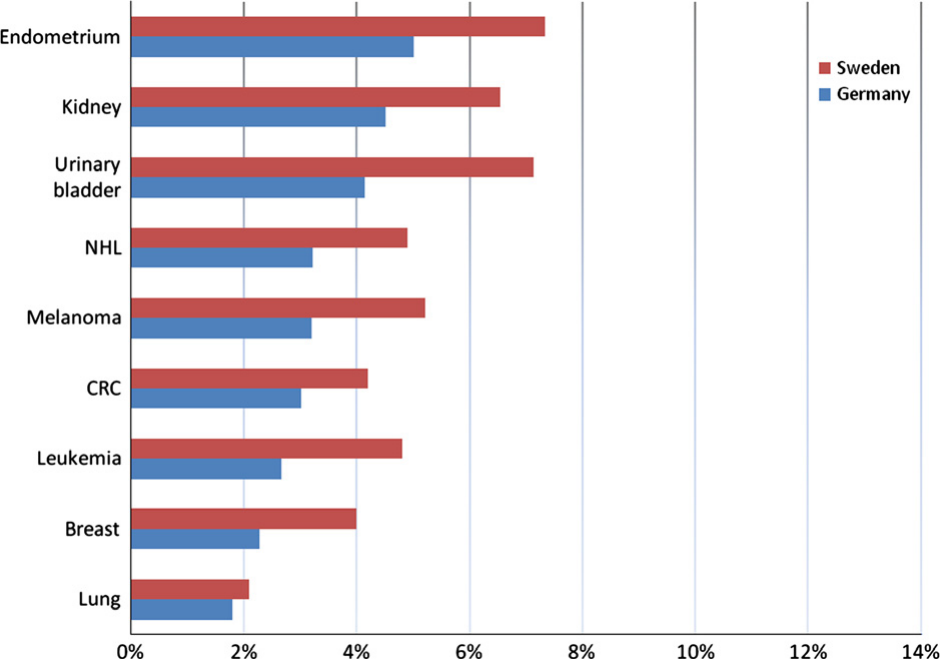
Percentage of all second primary cancers (SPCs) combined following 10 common first cancers in Germany and Sweden, female, 1997–2006.

Figures 1, 2 show percentage of all discordant SPCs combined following 10 common first cancers by sex in Germany and Sweden. The order of 10 common first cancers, ranked in descending incidence of the SPCs after the respective first cancers, was similar in the two populations, irrespective of the patients' gender. For instance, male patients with bladder cancers had the highest incidence of the SPCs (11.5% vs. 13.0%), and those with lung cancers had the lowest incidence of the SPCs (2.3% vs. 2.3%) (Fig. 1). For female patients, the highest SPC incidence appeared after endometrial cancers (5.0% vs. 7.4%) and the lowest one appeared after lung cancer (1.8% vs. 2.1%) in the two datasets (Fig. 2).

## Discussion

To our knowledge, this is the first comprehensive population-based comparison of SPCs in different populations. When comparing the ranking and proportion of the five most common SPCs after particular first primary cancers between the two populations, we found that there was a similar order of the five leading SPCs after most selected first primaries, especially for kidney cancer, NHL, and leukemia (Table 2). Furthermore, high positive correlations between the German and Swedish datasets were observed based on the ranks of thirty possible SPCs after all selected first cancers (Table 2). Of course the high correlation is driven by the overall incidence of cancers, including SPCs but nevertheless the consistent findings in the two populations provide support for the existence of uniform etiological processes underlying SPCs.

An important question in discussing similarities in the occurrence of SPCs is the incidence of first primary cancers. According to Cancer Incidence in Five Continents IX (http://www.iarc.fr), the male incidence of all cancers (around year 2000, without skin cancer) was 261 in Sweden (per 100,000) compared with 332 in Saarland, 345 in Mecklenburg-Western Pomerania, 315 in Munster, and 291 in Hamburg (as examples of the German rates, provided by these cancer registries). The differences were largely explained by tobacco-related sites because the incidence in lung cancer was almost three times higher in German men compared with Swedes. CRC was also more common in Germany while the opposite was true for prostate cancer; however, even for these cancers the Swedish rates were approximately within the incidence range in Germany. For women, the incidence of all cancer in Sweden was between the individual rates for the German cancer registries, and this applied to practically all individual sites.

Major strengths of this study are its design of population-based comparison and its subanalysis by specific SPCs. Analysis of large cancer registries including the Swedish Cancer Registry has consistently shown an excess burden of all SPCs combined, that is, there is an increased risk to develop any SPC after a primary cancer 11–17. However, the present analysis by specific SPCs showed a more thorough picture, in which the more detailed information may reveal a compelling clinical agenda. However, it is important to keep in mind that surveillance bias may contribute to an excess risk of SPCs. Chance findings are also possible but the consistent results for the two datasets should reduce such possibilities.

In this study, the correlation coefficients for SPCs after 10 common cancers other than bladder cancer (*r*_s_: 0.72) were greater than 0.80, indicating strikingly consistent patterns for developing a specific SPC in the two populations. Additionally, the five most common SPCs after kidney cancer, NHL, or leukemia were totally identical between the two datasets, and those after CRC, lung cancer, breast cancer, endometrial cancer, bladder cancer, or melanoma was 80% identical (4/5). The present findings showed that prostate cancer was the most common SPC in male cancer survivors with CRC, kidney cancer, bladder cancer, melanoma, or leukemia in the two populations, and breast cancer was the most common one after endometrial cancer. Although the detection of these SPCs may often be affected by general population screening such as screening mammography and prostate-specific antigen screening, aggregate data on SPCs and the specific risk data may certainly guide surveillance protocols tailored to the SPCs in question and inform decisions about personalized medicine. Taken together, the above consistent findings in the two populations should facilitate early detection and treatment of SPC 18.

State-of-the-art investigations on SPCs should also cover genetic predisposition 19. A recent genome-wide association study (GWAS) successfully explored the common genetic variations associated with susceptibility to SPCs 20. This study was conducted in survivors of Hodgkin's lymphoma treated with radiotherapy, and successfully identified a set of single nucleotide polymorphisms (SNPs) at chromosome 6p21 that were strongly associated with SPC risk. Such studies may shed light on the way for further investigations of the complex interactions between genetic and environmental factors involved in the development of SPC 21. Heterogeneity among the SPCs, however, was substantial, pointing out the need for separate evaluations by specific first and second primary pairs to explore the etiology of SPCs 22. A 2007 study showed a common genetic risk factor (Chr. 8q24 variants) for colorectal and prostate cancers 23. The present study supported the association between these two cancers at a population level and through replications in the two populations. The associations between first and SPCs may reflect emerging research priorities into SPC. For instance, there has been inconsistent evidence of excess bladder cancer after prostate cancer 24–27. A number of studies linked an increased risk of bladder cancer specifically to prostate cancer radiotherapy or smoking 28–30.

In summary, the consistent patterns of the SPCs in the German and the Swedish populations were striking. Results on SPCs provide guidance to surveillance and early intervention strategies which should be tailored to specific SPCs. Effective prevention of SPCs requires a better understanding of their causes.
